# A randomized trial of artesunate-amodiaquine *versus* artemether-lumefantrine in Ghanaian paediatric sickle cell and non-sickle cell disease patients with acute uncomplicated malaria

**DOI:** 10.1186/1475-2875-13-369

**Published:** 2014-09-19

**Authors:** George O Adjei, Bamenla Q Goka, Christabel C Enweronu-Laryea, Onike P Rodrigues, Lorna Renner, Abdul M Sulley, Michael Alifrangis, Insaf Khalil, Jorgen A Kurtzhals

**Affiliations:** Centre for Tropical Clinical Pharmacology and Therapeutics, University of Ghana Medical School, College of Health Sciences, Accra, Ghana; Department of Child Health, University of Ghana Medical School, College of Health Sciences, Accra, Ghana; Centre for Medical Parasitology at Department of International Health, Immunology and Microbiology, University of Copenhagen and Department of Clinical Microbiology and Department of Infectious Diseases, Copenhagen University Hospital (Rigshospitalet), Copenhagen, Denmark

**Keywords:** Sickle cell disease, Malaria, Children, Clinical trial, Artemisinin combination therapy, Ghana

## Abstract

**Background:**

Sickle cell disease (SCD) is a genetic disorder common in malaria endemic areas. In endemic areas, malaria is a major cause of morbidity and mortality among SCD patients. This suggests the need for prompt initiation of efficacious anti-malarial therapy in SCD patients with acute malaria. However, there is no information to date, on the efficacy or safety of artemisinin combination therapy when used for malaria treatment in SCD patients.

**Methods:**

Children with SCD and acute uncomplicated malaria (n = 60) were randomized to treatment with artesunate-amodiaquine (AA), or artemether-lumefantrine (AL). A comparison group of non-SCD children (HbAA genotype; n = 59) with uncomplicated malaria were also randomized to treatment with AA or AL. Recruited children were followed up and selected investigations were done on days 1, 2, 3, 7, 14, 28, 35, and 42. Selected clinical and laboratory parameters of the SCD patients were also compared with a group of malaria-negative SCD children (n = 82) in steady state.

**Results:**

The parasite densities on admission were significantly lower in the SCD group, compared with the non-SCD group (p = 0.0006). The parasite reduction ratio (PRR) was lower, clearance was slower (p < 0.0001), and time for initial parasitaemia to decline by 50 and 90% were longer for the SCD group. Adequate clinical and parasitological response (ACPR) on day 28 was 98.3% (58/59) in the SCD group and 100% (57/57) in the non-SCD group. Corresponding ACPR rates on day 42 were 96.5% (55/57) in the SCD group and 96.4% (53/55) in the non-SCD group. The fractional changes in haemoglobin, platelets and white blood cell counts between baseline (day 0) and endpoint (day 42) were 16.9, 40.6 and 92.3%, respectively, for the SCD group, and, 12.3, 48.8 and 7.5%, respectively, for the non-SCD group. There were no differences in these indices between AA- and AL-treated subjects.

**Conclusions:**

The parasite clearance of SCD children with uncomplicated malaria was slower compared with non-SCD children. AA and AL showed similar clinical and parasitological effects in the SCD and non-SCD groups. The alterations in WBC and platelet counts may have implications for SCD severity.

**Trial registration:**

Current controlled trials ISRCTN96891086.

## Background

Sickle cell disease (SCD) is the most common monogenic disease worldwide. SCD is an autosomal recessive condition that results from substitution of glutamic acid with valine in the sixth position of the beta-globin chain of haemoglobin. SCD occurs in individuals homozygous for the β^S^ globin gene (SS) or in individuals heterozygous for the β^S^ allele and other abnormal β globin gene alleles such as β^C^ (SC), S β^0^ thalassemia, or Sβ^+^ thalassemia. SCD is associated with significant morbidity and mortality and is characterized clinically among others, by chronic haemolysis, vaso-occlusive events and increased susceptibility to infections. The highest frequencies of homozygous SCD are found in sub-Saharan Africa, where intense malaria transmission occurs.

In endemic areas, malaria is considered a major cause of the morbidity and mortality that SCD subjects experience. A linkage exists between the presence of sickle haemoglobin (HbS) and protection from malaria in the heterozygous state [1–3]; however, the homozygous (HbSS) state may be associated with increased susceptibility to malaria [4, 5]. Malaria has been cited as a precipitating cause of acute crises in adults with SCD [6], and as a major cause of frequent hospitalization [7], as well as poor outcome [8] among children with SCD. Even though evidence from recent studies indicates that overall prevalence of malaria in SCD subjects is comparatively low, it has been shown that among hospitalized SCD patients mortality is higher among those with malaria compared with those without [9–14]. These considerations suggest that prompt initiation of efficacious anti-malarial therapy among acutely ill SCD patients with confirmed malaria is important. However, there is paucity of information on the effects of anti-malarial treatment in SCD patients with acute malaria in general, and no report to date on the efficacy or safety of currently recommended first-line malaria treatments, artemisinin combination therapy (ACT), when used for treatment in SCD patients with malaria.

It is possible that response to anti-malarial therapy may differ between SCD and non-SCD subjects undergoing anti-malarial treatment. Indeed, plasma chloroquine concentrations have been shown to be 2-3 fold lower in subjects with SCD (HbSS) compared to those with HbAA or HbAS, and chloroquine accumulation has been shown to differ between SCD and non-SCD erythrocytes *in vitro* [15]. Platelets and leucocytes have also been shown to accumulate large amounts of chloroquine [16], suggesting that conditions, such as SCD, that are associated with thrombocytosis or leucocytosis, may influence anti-malarial drug distribution and consequently, effective plasma concentrations. Furthermore, known pathophysiological changes that are prevalent in SCD patients such as malnutrition and hypoalbuminaemia [17–19], could also alter anti-malarial drug distribution and metabolic clearance, which may influence the disposition of selected drugs in patients with these changes.

In the present study, the efficacy of artesunate-amodiaquine (AA) and artemether-lumefantrine (AL), the two most commonly adopted ACT regimens across sub-Saharan Africa, were evaluated among SCD children with parasitologically confirmed acute uncomplicated malaria in Accra, Ghana. Selected haematological parameters in treated SCD children were compared with those of non-SCD children (HbAA genotype), as well as with comparable haematological parameters of a group of apparently well, microscopically and malaria rapid diagnostic test (RDT)-negative SCD children in steady state.

## Methods

### Study site

The study was conducted at the Outpatients Department (OPD) and Paediatric Sickle Cell Clinic of the Department of Child Health (DCH), Korle Bu Teaching Hospital (KBTH), between January 2010 and December 2011. The KBTH is a 1,600-bed tertiary referral hospital in Accra, Ghana. Malaria transmission occurs all year in Ghana, peaking in July-August immediately after the major rainfall season. The prevalence of malaria parasitaemia in Accra ranges between 6 and 22% (average 14.8%) depending on the community [20]. The sickle cell trait occurs in up to 25% of the population [21] and an SCD prevalence of 2% has been reported among newborns in Ghana [22].

The DCH is a referral centre within KBTH for children aged less than 13 years with acute or chronic medical and surgical conditions. The OPD is the first point of call for referred cases to the DCH. The OPD has an average yearly attendance of 36,000, and is divided into an acute care outpatients and specialty clinics. The Paediatric Sickle Cell Clinic is one of the specialty clinics at the DCH and has about 5,000 SCD patients below 13 years of age registered. The Paediatric Sickle Cell Clinic provides routine and follow-up care for children with SCD. The Clinic is held once a week and has an average weekly attendance of about 60-70. Aside from the Clinic, SCD children with acute illness are seen at the Paediatric Emergency Department 24 hours a day throughout the week.

### Study population and design

Children of known SCD status registered at the Paediatric Sickle Cell Clinic, who presented to the Paediatric Emergency Department, or the Paediatric Sickle Cell Clinic with an acute febrile illness were referred to the study team. A study physician examined the patient and recruited subjects if study criteria were fulfilled. At the adjoining primary care facility (Korle Bu Polyclinic) children not known to have SCD presenting with an acute febrile illness were also referred to the study team and recruited if study criteria were fulfilled. A group of known SCD children visiting the Paediatric Sickle Cell Clinic for routine scheduled follow-up visits, who were determined to be in steady state on the day of visit (n = 82), were also recruited for the purpose of comparing selected haematological indices between malaria-positive and malaria-negative SCD children.

### Criteria for inclusion

Inclusion criteria for the study were: child of confirmed SCD status aged six months to 12 years with an acute febrile illness (history of fever within the previous 72 hours, or an axillary temperature ≥37.5°C at presentation); *Plasmodium falciparum* infection of parasite density <200,000/μL; and, willingness of the accompanying parent/guardian to comply with the study procedures and follow-up schedule. Exclusion criteria were: symptoms or signs of severe malaria; known intolerance or allergy to study medications; and, reported treatment with any of the study drugs one month preceding enrolment. The inclusion and exclusion criteria for the non-SCD (HbAA) children were the same as for the SCD children apart from haemoglobin genotype. The SCD children in steady state were children with confirmed SCD status registered at the Paediatric Sickle Cell Clinic who were asymptomatic and were visiting the Clinic for routine scheduled evaluations. These children had a negative blood film and negative malaria RDT result on the day of recruitment. A standardized evaluation was done for assessing and recording presenting symptoms and clinical signs and for recording initial laboratory investigations.

### Randomization and treatment allocation

A computer-generated simple randomization scheme was prepared in advance. Allocated treatments were kept in sealed, opaque envelopes which were opened after completion of all formal enrolment procedures and were then administered.

### Treatment administration

Artesunate-amodiaquine (Coarsucam^®^, Sanofi Aventis, France; 25/50/100 mg artesunate and 67.5/135/270 mg amodiaquine), single daily dose, was administered for three days according to body weight: ≥4.5- < 9 kg (25 mg/67.5 mg), one tablet/dose; ≥9- < 18 kg (50 mg/135 mg), one tablet/dose; ≥18- < 36 kg (100 mg/270 mg), one tablet/dose; ≥36 kg (100 mg/270 mg), two tablets/dose. Artemether-lumefantrine (Coartem^®^, Novartis Pharma AG, Basel, Switzerland; 20 mg artemether and 120 mg lumefantrine) was administered at zero and eight hours on the first day, and then twice daily for two subsequent days according to body weight: 5-14 kg, one tablet/dose; 15-24 kg, two tablets/dose; 25-34 kg, three tablets/dose; 35 kg and over, four tablets/dose. All drug doses were administered by study nurses at the clinic. At the clinic, children were observed for one hour after each drug administration. Treatments were re-administered if the child vomited within the observation period. Children who vomited the re-administered dose were withdrawn.

### Follow-up

Recruited subjects were followed up on days 1, 2, 3, 7, 14, 28, 35, and 42 and on any other day outside the scheduled follow-up days if they became ill or had any health concerns. Children who missed their scheduled day 14, 28 or 42 follow-up appointments were seen on days 15, 29 or 43, respectively. Follow-up assessments consisted of a standardized evaluation for assessing persistence of presenting symptoms or clinical signs (or for new/emergent symptoms or signs), physical examination, and laboratory investigations, described below.

### Laboratory investigations

Venous blood was collected into EDTA and heparinized sample tubes on days 0, 3, 7, 14, 28, and 42. On follow-up days 1 and 2, only a finger prick sample for a blood smear for malaria microscopy was done. Malaria parasitaemia (expressed as parasites per microlitre of whole blood) was determined in Giemsa-stained thick blood films. Parasite density was determined by counting the number of asexual stage parasites relative to 200 white blood cells (WBC), and multiplied by the measured WBC count. Each slide was read independently by two microscopists and the average of two readings recorded. Approximately 10% of all slides were read by a third expert microscopist who was independent of the study. Any discrepancies in the readings were checked with all three microscopists present and a consensus reached. RDT for the histidine-rich protein 2 (First Response^®^, Premier Medical Corporation Ltd, India) and parasite lactic dehydrogenase (Optimal^®^, Diamed, Basel, Switzerland) were done according to the manufacturers’ instructions. The total WBC and differential counts were measured by means of an automated haematology analyzer (Sysmex KX-21 N, Sysmex Inc, USA). Clinical chemistry parameters were measured, using a chemistry analyzer (Vitros^®^ Systems, Ortho Clinical Diagnostics, USA). A nested PCR method was used to analyse polymorphisms in the genes coding for the merozoite surface protein (MSP) 1 and 2 to distinguish between recrudescent and new infections [23, 24]. The plasma concentrations of amodiaquine and its metabolites (desethylamodiaquine; bis-desethylamodiaquine) and lumefantrine and its metabolites (desbutyllumefantrine) were measured by means of a high performance liquid chromatographic (HPLC) method [25]. The SCD or HbAA status of recruited subjects was determined by a sodium metabisulphite test and alkaline haemoglobin electrophoresis.

### Endpoint assessment

The primary endpoint was the parasite clearance rate on days 1, 2 and 3 of treatment, determined by the parasite reduction ratios (PRR), and the time for initial parasitaemia to decline by 50% (PC_50_), and by 90% (PC_90_).

The rationale for choosing a primary parasitological endpoint was based on results from previous studies, which have shown consistently high (>95%) overall cure rates (on day 28 or 42) after treatment with either AA or AL [26–28]. These high cure rates for both ACT regimens make it unlikely that a clinically important difference would be detected between the two ACT regimens on day 28 or 42. However, as indicated, underlying disease may influence the disposition of anti-malarial drugs among SCD and non-SCD patients. These may influence effective plasma concentrations and possibly treatment response in the initial days (1-3) of treatment.

Other outcome measures included parasitaemia recurrence over the follow-up period after initial clearance and changes in selected haematological profiles in the respective groups during follow-up. In the context of SCD, the extent of changes in parameters such as haemoglobin, leucocytes, neutrophils, and platelets could have important ramifications for disease-related morbidity. Other secondary outcome measures included day 28 and 42 cure rates, classified as early treatment failure (ETF), late clinical failure (LCF), late parasitological failure (LPF), or adequate clinical and parasitological response (ACPR), according to the WHO 2005 guidelines [29].

Adverse events were defined as any untoward medical occurrence, irrespective of its suspected relationship to study medications according to International Conference on Harmonization (ICH) guidelines.

### Statistical analyses

The study was designed to detect a difference in parasite clearance and fractional reduction in parasite count (PRR; PC_50_; PC_90_) between the SCD and non-SCD groups. A sample size of 27 per group was sufficient to detect, at 90% power and 95% confidence, a 30% difference in parasite clearance, or at least a two-fold change in PRR between the SCD and non-SCD groups on day 2. The PRR was defined as the ratio of the parasite count before treatment to the parasite count on days 1, 2 or 3. Time to 50 and 90% parasite clearance were obtained from the intercept on a plot of parasitaemia *versus* time. A random-effect generalized least square regression method was used to compare parasite densities between the SCD and non-SCD groups on successive days. The parasite clearance rates on the specific days were also compared, using survival analysis. Categorical variables were compared, using the Chi square test with Yates’s correction, or the Fisher’s exact test as appropriate. Continuous variables were compared, using the student’s t-test, or one-way analysis of variance, or the Kruskall-Wallis test, as appropriate. For the survival analysis, subjects who did not complete 42 days of follow-up were censored on their last follow-up date. Probability values <0.05 were considered significant. Data were analysed using Stata™ (Version 10).

### Ethics

Ethical approval for the study was granted by the Ethical and Protocol Review Committee of the University of Ghana Medical School. Written, informed consent was obtained from the parents or guardians of all enrolled children.

## Results

### Baseline characteristics

**Figure 1:**
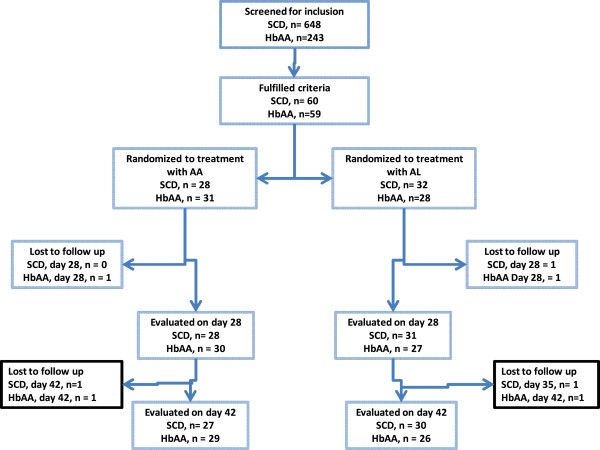
**Trial profile.** SCD = sickle cell disease; HbAA = non-SCD; AA = artesunate-amodiaquine; AL = artemether-lumefantrine.

A total of 648 SCD children presenting with acute febrile illness at the OPD and Paediatric Sickle Cell Clinic, DCH, were screened for inclusion. Of those screened, 60 subjects with confirmed *P. falciparum* malaria fulfilled inclusion criteria and were randomized to treatment with AA (n = 28), or AL (n = 32). The SCD genotype distributions among the recruited subjects were: SS, n = 43 (71.66%); SC, n = 11 (18.33%); SS/SD, n = 5 (8.3%); and, Sβthal, n = 1 (1.6%). A total of 243 non-SCD (HbAA) children presenting with an acute febrile illness were screened at the Korle Bu Polyclinic for inclusion, out of which 59 with confirmed *P. falciparum* uncomplicated malaria were randomized to treatment with AA (n = 31) or AL (n = 28). The flow of subjects through the study is shown (Figure 1). A total of 82 SCD children in steady state presenting to the Paediatric Sickle Cell Clinic for their scheduled routine evaluation visits and with a negative blood film as well as negative RDT test results were recruited.

### Admission parameters

The baseline admission clinical and laboratory parameters were significantly different between the malaria positive-SCD and malaria positive-non-SCD (HbAA) groups (Table 1). Within the SCD group, the mean baseline admission characteristics between AA and AL treated subjects (age [years], 7.71 *vs* 7.60; weight [kg], 22.80 *vs* 23; temperature [°C], 37.83 *vs* 38.09; parasite density [/μL], 1,012 *vs* 1,408; haemoglobin [g/dL], 6.71 *vs* 6.40; WBC [×10^3^ L], 23.87 *vs* 24.04; and platelets [×10^3^L], 263.11 *vs* 246.56) were not statistically significant. Selected characteristics of the SCD-steady state subjects were significantly different from that of the malaria-positive SCD patients (Table 2).

**Table 1:** **Admission characteristics of sickle cell disease (SCD) or non-SCD (HbAA) subjects with uncomplicated malaria**

	SCD (n = 60)	HbAA (n = 59)	p
Age* (years)	7.92 (6.98-9.98)	5.45 (4.67-6.35)	0.001
Weight (kg)	23.18 (19.11)	20.54 (8.34)	0.077
Temperature (°C)	37.90 (1.12)	38.62 (1.12)	0.001
Jaundice (n)	42 (79.2 %)	4 (6.7 %)	< 0.001
Spleen size (cm)	1.27 (2.73)	0.20 (0.81)	0.008
Liver size (cm)	3.42 (4.70)	0.43 (1.32)	< 0.001
Parasite density* (/μL)	1,197 (645-2221)	11,774 (5256-26375)	0.0006
Haemoglobin (g/dL)	6.56 (2.22)	9.71 (2.16)	<0.0001
WBC (10^9^/L)	23.95 (18.45)	10.21 (5.92)	<0.0001
Platelet count (10^9^/L)	254.8 (133.9)	154.9 (106.1)	<0.0002

Data are means (SD), except *geometric mean (95% CI), SCD = sickle cell disease; HbAA = non-SCD.

**Table 2:** **Admission characteristics of sickle cell disease (SCD) subjects with malaria** ***versus*** **that of SCD subjects in steady state**

	SCD-malaria (n = 60)	SCD Steady state (n = 82)	p
Age* (years)	7.92 (6.98-9.98)	6.86 (6.15-7.65)	0.072
Weight (kg)	23.18 (19.11)	-	-
Temperature (°C)	37.90 (1.12)	-	-
Jaundice (n)	42 (79.2 %)	-	-
Spleen size (cm)	1.27 (2.73)	-	-
Liver size (cm)	3.42 (4.70)	-	-
Parasite density* (/μL)	1,197 (645-2,221)	-	-
Haemoglobin (g/dL)	6.56 (2.22)	8.14 (1.59)	<0.0001
WBC (10^9^/L)	23.95 (18.45)	13.76 (4.34)	<0.0001
Platelet count (10^9^/L)	254.8 (133.9)	413.122 (184.38)	<0.0001

Data are means (SD), except *geometric mean (95% CI), SCD = sickle cell disease; SCD Steady state = sickle cell disease (malaria-negative) subjects in steady state.

### Presenting symptomatology

The predominant presenting symptom for both groups was a history of fever. However, the proportion of SCD subjects presenting with elevated temperature at admission was comparatively low. The remaining presenting symptoms among the SCD subjects were mostly vaso-occlusive complex-related (Table 3). The reported duration of symptoms of subjects in the SCD group ranged from one to eight days (mean (SD), 2.72 (1.67)), while reported symptom duration among the non-SCD group ranged between one and 14 days (mean (SD) 3.60 (2.67)).

**Table 3:** **Frequency of presenting symptoms and signs among sickle cell disease (SCD) and non-SCD (HbAA) children with uncomplicated malaria**

Symptom/sign	SCD (n = 60)	Symptom/sign	HbAA (n = 59)
Fever*	33	Fever	57
Bone and/or joint pain	24	Vomiting	32
Abdominal pain	11	Headache	21
Headache	10	Abdominal pain	17
Chills and/or rigors	9	Poor appetite	11
Jaundice	7	Cough	6
General malaise	5	General malaise	4
Vomiting	4	Convulsion	3
Dark urine	4	Nasal congestion	2
Poor appetite	3	Chills and/or rigors	1
Cough	3	Diarrhoea	1
Dizziness	2	Dizziness	1
Easy fatigability	2	Weight loss	1
Sore throat	1	-	
Nasal congestion	1	-	
Priapism	1	-	
Swollen lower limb	1	-	

*Fever = measured temperature ≥ 37.5°C; majority of subjects presented with more than one symptom; therefore, data is not 100% cumulative.

### Parasite clearance: SCD *versus*non-SCD groups

**Figure 2:**
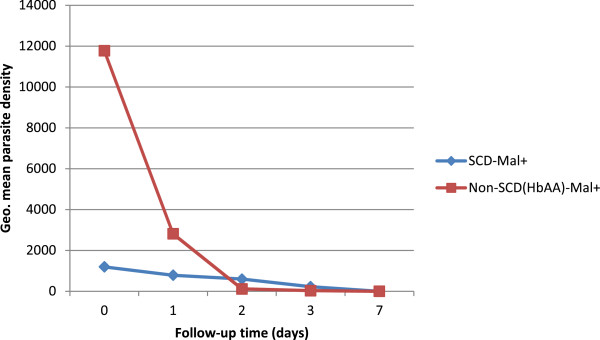
**Parasite clearance** ***versus*** **time (days) in the sickle cell disease (SCD) and non-SCD (HbAA) groups.** SCD-mal + = sickle cell disease subjects with acute malaria group; Non-SCD (HbAA)-mal + = non-SCD subjects with acute malaria group.

**Figure 3:**
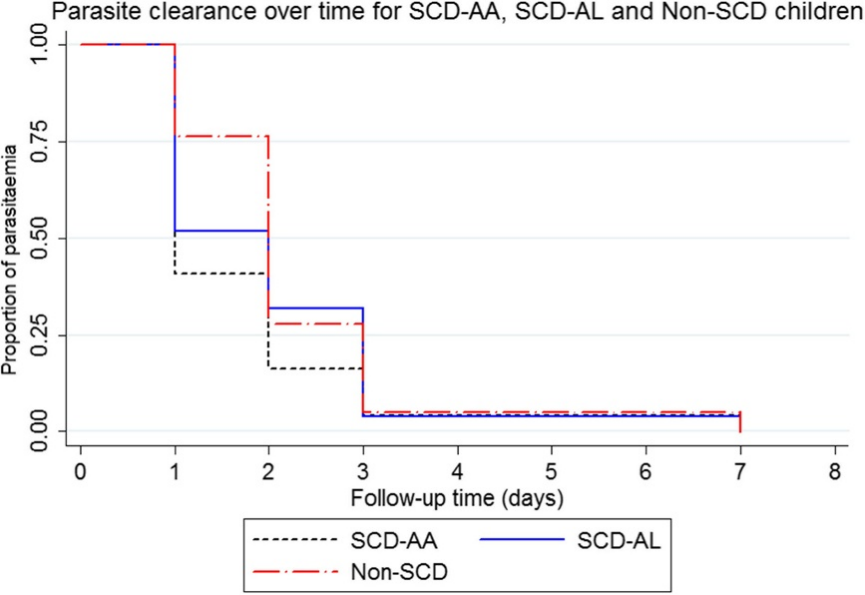
**Survival analysis showing parasite clearance per individual in the respective groups.** SCD-AA = artesunate-amodiaquine-treated sickle cell disease subjects with acute malaria; SCD-AL = artemether-lumefantrine-treated sickle cell disease subjects with acute malaria; non-SCD = non sickle cell disease (haemoglobin genotype AA) subjects with acute malaria.

The parasite clearance of the SCD subjects appeared slower, compared with the non-SCD group (Figure 2). A random-effect generalized least squares regression analysis showed the clearance rate of the SCD group to be significantly different from the non-SCD group (Wald p-value < 0.0001). The PRR was lower, and the times for parasitaemia to decline, (by 50 and 90%) as determined from the intercept of the parasite clearance curve, was longer for the SCD group compared with the non-SCD group (Table 4). However, using Cox regression analysis, individual parasite clearance rate per day was 0.536 (95% CI, 0.408-0.704) among the SCD subjects compared with, 0.448 (95% CI, 0.328-0.614) among the non-SCD subjects (Mantel Haenszel rate ratio, 0.836, 95% CI, 0.552-1.267, p = 0.39). Similar results were obtained, using survival analysis (log rank test for equality of survival functions, p = 0.10, Figure 3).

**Table 4:** **Parasite reduction ratios in the various groups**

Group	Day of treatment	Parasite density (geometric mean)	PRR	Time to 50 % parasite density (days)	Time to 90 % parasite density (days)
**SCD**	Day 0	1197	-		-
	Day 1	788	1.52	2	5
	Day 2	599	1.99		
	Day 3	226	5.28		
**HbAA**	Day 0	11,774	-		
	Day 1	2,811	4.18	0.6	1.6
	Day 2	118	99.23		
	Day 3	32	367.95		
**SCD-AA**	Day 0	1,012	-		
	Day 1	684	1.47	2.7	3
	Day 2	2,135	0.47		
	Day 3	64	15.81		
**SCD-AL**	Day 0	1,408	-		
	Day 1	869	1.62	1.25	7
	Day 2	228	6.15		
	Day 3	2840	0.49		

SCD = sickle cell disease, HbAA = non-SCD, SCD-AA = SCD subjects treated with artesunate-amodiaquine, SCD-AL = SCD subjects treated with artemether-lumefantrine.

### Parasite clearance: AA *versus*AL among SCD subjects

Within the SCD group, the parasite clearance rate per day was 0.58 (95% CI, 0.393-0.849) for the AA-treated group, compared with 0.50, (95% CI, 0.340-0.734) for the AL-treated group (Mantel-Haenszel rate ratio, 0.865, 95% CI, 0.502-1.492, p = 0.60). Similar results were obtained, using survival analysis (log rank test for equality of survival functions, p = 0.30, Figure 3).

### Cure rates on day 28 and 42: SCD *versus*non-SCD groups

The proportion of evaluable subjects with uncorrected ACPR on day 28 was 98.3% (58/59) in the SCD group, and 100% (57/57) in the non-SCD group, respectively. The corresponding ACPR rates on day 42 were 96.5% (55/57) in the SCD group and 96.4% (53/55) in the HbAA group. The PCR-corrected ACPR rates on day 42 were 98.2% (56/57) in the SCD group and 98.2% (54/55) in the non-SCD group (Table 5).

**Table 5:** **Efficacy outcomes on day 28 and 42 in the SCD and HbAA groups**

Day 28	SCD		HbAA	
	Uncorrected	PCR-corrected	Uncorrected	PCR-corrected
ACPR	58	59	57	57
ETF	0	0	0	0
LCF	0	0	0	0
LPF	1	0	0	0
**Day 42**	**SCD**		**HbAA**	
ACPR	55	56	53	54
ETF	0	0	0	0
LCF	0	0	0	0
LPF	2	1	2	1

ACPR = adequate clinical and parasitological response, ETF = early treatment failure, LCF = late clinical failure, LPF = late parasitological failure, SCD = sickle cell disease, HbAA = non-SCD.

### Cure rates on day 28 and 42: AA *versus*AL groups among SCD subjects

Within the SCD group, the proportion of evaluable AA-treated subjects with ACPR (uncorrected) was 96.4% (27/28) on day 28 and 92.6% (25/27) on day 42. The corresponding ACPR rates in the non-SCD group were 100% (30/30) on day 28 and 100% (29/29) day 42. The proportion of AL-treated subjects with ACPR in the SCD group was 100% (31/31) on day 28 and 100% (30/30) on days 28 and 42, respectively. The corresponding ACPR rates in the non-SCD group were 100% (27/27) on day 28 and 92.3% (24/26) on day 42, respectively. Selected characteristics of children with recurrent parasitaemia during follow-up are shown (Table 6).

**Table 6:** **Selected characteristics of subjects with recurrent parasitaemia in the SCD and non-SCD groups**

Age (yrs)	Genotype	Admission parasitaemia	Day of recurrence	Treatment received	PCR result	Day 7 drug levels
9	SS	640	21	AA	New infection	DQ-21 ng/ml
4	SS/SD	2480	35	AA	Recrudescence	DQ-20 ng/ml
2.5	HbAA	79520	42	AL	Recrudescence	DL- ND
7	HbAA	63400	42	AL	New infection	DL - ND

SS = sickle cell anaemia, HbAA = non-SCD, AA = artesunate-amodiaquine, AL = artemether-lumefantrine, DQ = desethylamodiaquine, DL = desbutyllumefantrine, ND = not done.

### Selected haematological parameters on admission and during follow-up

**Figure 4:**
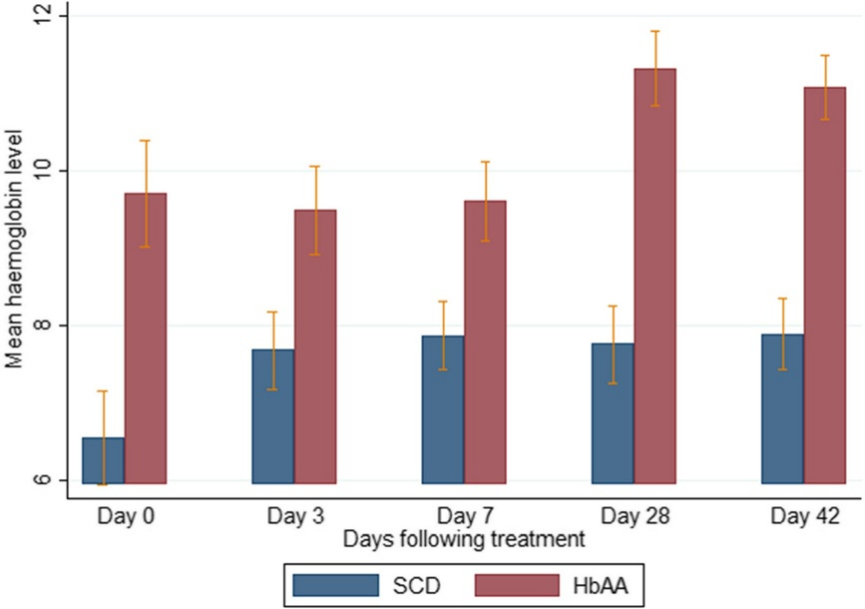
**Haemoglobin levels on admission and follow up between the sickle cell disease and non-sickle cell disease subjects.** Data are means and 95% confidence intervals (error bars). SCD = sickle cell disease subjects; HbAA = non-sickle cell disease subjects.

**Figure 5:**
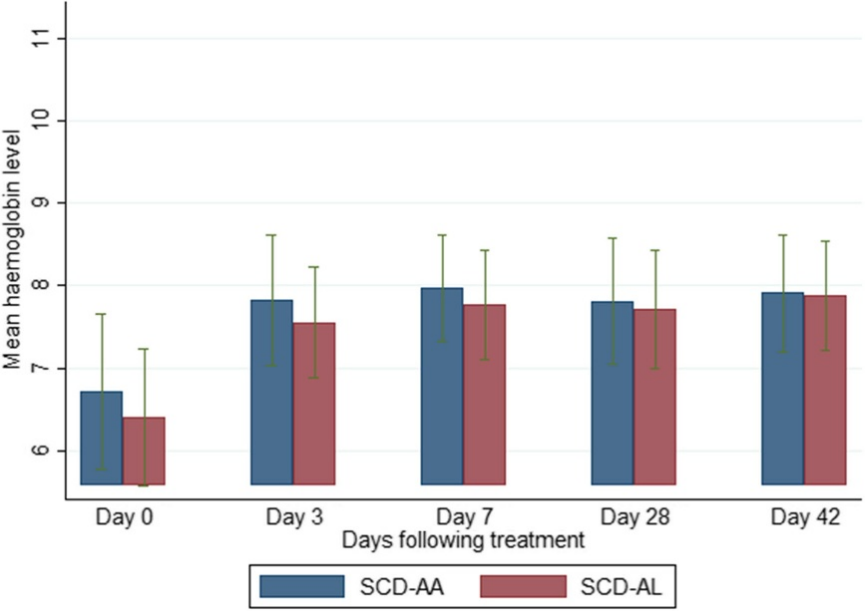
**Haemoglobin levels on admission and follow-up between the artesunate-amodiaquine- and artemether-lumefantrine-treated sickle cell disease subjects.** Data are means and 95% confidence intervals (error bars). SCD-AA = artesunate-amodiaquine-treated sickle cell disease subjects; SCD-AL = artemether-lumefantrine-treated sickle cell disease subjects.

**Figure 6:**
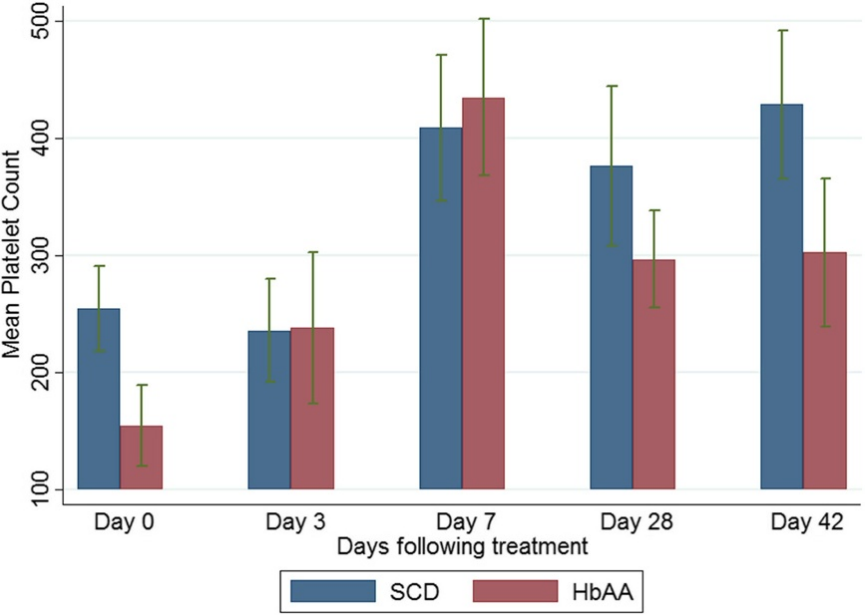
**Platelet counts on admission and follow-up between the sickle cell disease and non-sickle cell disease subjects.** Data are means and 95% confidence intervals (error bars). SCD = sickle cell disease subjects; HbAA = non-sickle cell disease subjects.

**Figure 7:**
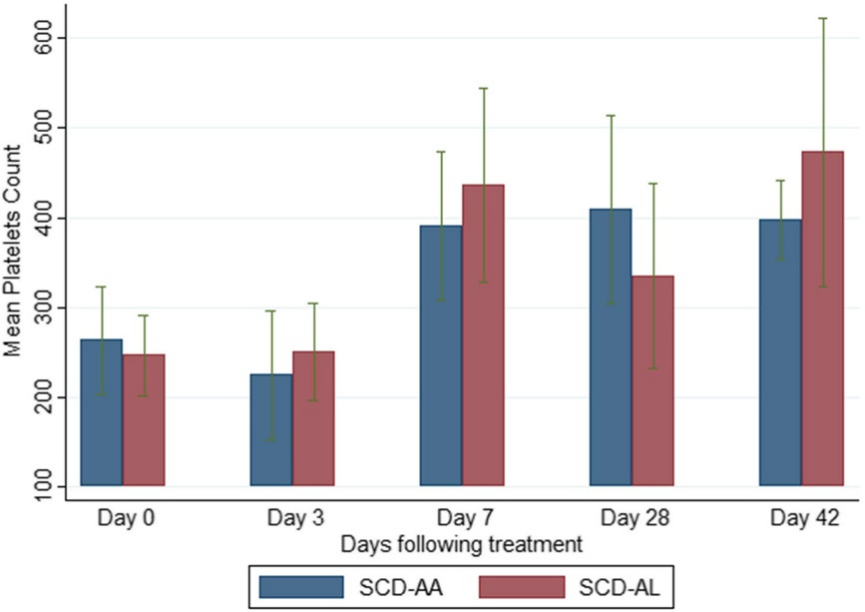
**Platelet counts on admission and follow-up between the artesunate-amodiaquine- and artemether-lumefantrine-treated sickle cell disease subjects.** Data are means and 95% confidence intervals (error bars). SCD-AA = artesunate-amodiaquine-treated sickle cell disease subjects; SCD-AL = artemether-lumefantrine-treated sickle cell disease subjects.

**Figure 8:**
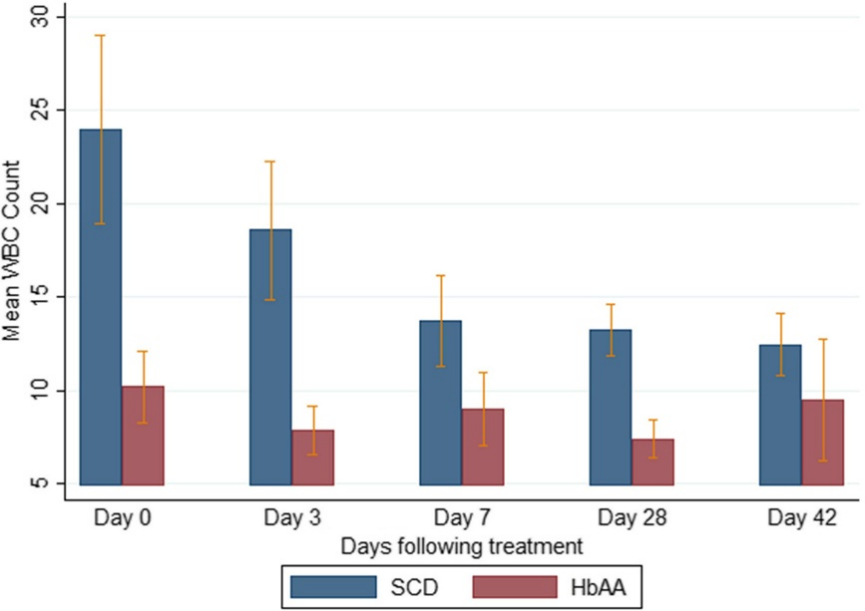
**Total white blood cell counts on admission and follow-up between the sickle cell disease and non-sickle cell disease subjects.** Data are means and 95% confidence intervals (error bars). SCD = sickle cell disease subjects; HbAA = non-sickle cell disease subjects.

**Figure 9:**
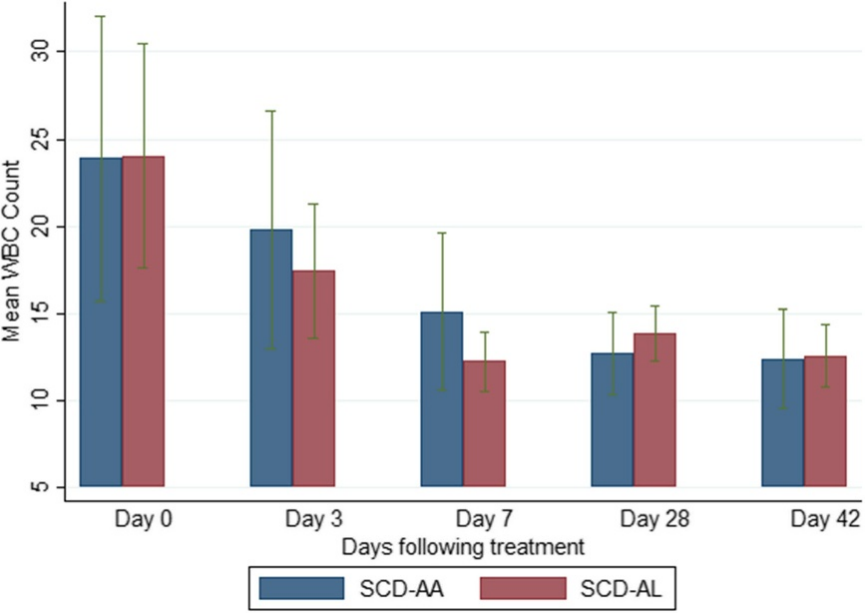
**Total white blood cell counts on admission and follow-up between the artesunate-amodiaquine- and artemether-lumefantrine-treated sickle cell disease subjects.** Data are means and 95% confidence intervals (error bars). SCD-AA = artesunate-amodiaquine-treated sickle cell disease subjects; SCD-AL = artemether-lumefantrine-treated sickle cell disease subjects.

The mean haemoglobin levels of the SCD-malaria positive subjects were lower than those of the non-SCD (HbAA)-malaria-positive subjects on admission and all scheduled follow-up days (Figure 4). Among the SCD subjects, the mean haemoglobin levels of the AA-treated and AL-treated subjects were similar (Figure 5). The platelet counts on admission and follow-up were significantly higher (except on days 3 and 7) in the SCD-malaria-positive subjects compared with the non-SCD (HbAA) malaria-positive subjects (Figure 6). Among the SCD subjects, the platelet counts on admission and follow-up were similar for the AA-treated and AL-treated subjects (Figure 7). The total WBC levels of the SCD-malaria-positive subjects on admission and follow-up were higher than those of the non-SCD subjects (Figure 8). Among the SCD patients, total WBC counts on admission and follow up were similar for the AA-treated and AL-treated subjects (Figure 9). The platelet counts of the SCD-steady state subjects were significantly higher than those of the SCD-malaria positive subjects on admission while the admission WBC count of the SCD-malaria patients was higher than those of the SCD steady state subjects (Table 2).

### Haematological index changes

The fractional change differences in the selected haematological indices (WBC, platelets, haemoglobin) between admission (day 0, acute illness) and endpoint (day 42, recovery/convalescence) in the SCD and non-SCD groups were WBC (92.3%, (SCD) *versus* 7.5%, (non-SCD); platelets (40.6%, (SCD) *versus* 48.8%, (non-SCD); and, haemoglobin (16.9%, SCD *versus* 12.3%, (non-SCD), respectively.

### Plasma desethylamodiaquine (DEAQ) concentrations in those with recurrent parasitaemia

The individual plasma DEAQ concentrations in the two AA-treated subjects who experienced recurrent parasitaemia were 20 ng/ml and 21 ng/ml, respectively.

### Adverse events

Adverse events emerging during and after treatment overlapped substantially with known malaria or SCD symptomatology, could not be attributed to administered treatments, and were in most cases adjudged as unrelated to study medications.

## Discussion

This study reports treatment response of paediatric SCD patients with acute uncomplicated malaria to recommended first-line ACT regimens. In particular, the study reports for the first time systematically collected data on parasite clearance and on selected haematological parameters (haemoglobin, WBC, platelets) in SCD children undergoing ACT treatment.

The data showed significantly lower parasite densities on admission, slower clearance dynamics, lower PRR values, and longer time to 50 and 90% parasite clearance in the SCD, compared with the non-SCD children. The significantly lower parasite densities among SCD patients compared with non-SCD patients has been reported in previous studies [2, 30, 31]. This is thought to result from the protective effect of the sickle haemoglobin [3, 32, 33], via mechanisms that include reduced parasite growth [34], enhanced phagocytosis [35, 36], and removal of parasitized cells - through innate or acquired immunological processes [2]. The degree of protection conferred by the sickle phenomenon has been shown to correlate with intracellular HbS concentration implying a greater propensity to protection in homozygous subjects. The protective effect of the sickle haemoglobin on malaria has also been ascribed to aberrant host actin remodelling [37], and to accelerated breakdown of haem oxygenase, which is strongly induced by the sickle haemoglobin [38]. More recently, the role of dysregulated microRNA activity, where growth inhibitory host microRNAs are translocated into the parasite, or fuse with extant parasite mRNA transcripts to inhibit translation of enzymes critical for parasite development has also been described [39].

The lower PRR and longer time to 50 and 90% parasite clearance among the SCD-malaria patients compared with non-SCD malaria patients has not been previously reported. This is partly because there have been no previous studies reporting parasite clearance changes among SCD patients undergoing anti-malarial treatment. The apparent disparity between the slower clearance, lower PRR’s and time to 50 and 90% clearance of the SCD *versus* non-SCD group data on one hand, and the comparable clearance rates between the two groups from the Cox regression analysis may reflect a convergence effect due to the significant initial parasitaemia differences between the two groups. The several-fold difference in admission parasite density between the two groups makes direct comparison problematic. However, the lower PRR and clearance rate in the SCD group in spite of a lower initial parasitaemia suggests a true between-group difference. The cure rates on day 28 or 42 were high for both the SCD and non-SCD groups. This is consistent with the high efficacy rates for AA and AL that have been reported across sub-Saharan Africa [26–28, 40]. Indeed, AA and AL have both been associated with high day 28 cure rates when used for treatment of uncomplicated malaria, even among children with underlying immune-deficiency [41–43]. It is reasonable to expect therefore, that the lower PRR in the SCD group is unlikely to translate overall into difficulties with treatment. However, the implications of this finding to symptom resolution and malaria-related morbidity in SCD patients are unclear.

The individual plasma drug concentrations of DEAQ (20 ng/ml; 21 ng/ml) in the two AA-treated subjects with recurrent parasitaemia were lower than the mean plasma concentrations (115 ng/ml; 156.6 ng/ml) [44, 45] that has been reported for (non-SCD) children treated with these regimens. It has been suggested that an *in vivo* day 3 DEAQ concentration of 135 ng/ml is the minimum level required for adequate treatment efficacy [45]. This suggests that these cases of recurrent parasitaemia are likely due to inadequate exposure, rather than parasite resistance. It cannot also be ruled out that low plasma DEAQ concentrations may result from variable disposition of these anti-malarials among SCD patients. This may suggest that pharmacokinetic studies of ACT regimens in populations with other significant co-morbidities may be warranted.

The lower fever incidence and higher WBC counts in the SCD group at presentation may present specific difficulties, especially with respect to diagnosis of diseases of bacterial origin. However, a raised WBC in SCD is also a recognized risk factor for complications ranging from acute chest syndrome, silent cerebral infarction, clinically overt stroke, and early death [46–52]. In this respect, the raised WBC at the acute illness stage among the SCD patients may represent an additional (malaria-related) prognostic indicator.

The majority of SCD subjects presented on admission with symptoms and signs consistent with vaso-occlusive complex (VOC) -related phenomena. Vaso-occlusion crisis is a hallmark of SCD but the mechanism(s) responsible for triggering VOC in SCD is likely a result of complex interplay of precipitating factors at multiple stages. However, it has been shown that SCD subjects in VOC have higher levels of pro-inflammatory cytokines compared with those in steady state [53]. Since malaria has been shown to be a precipitating factor for VOC in SCD patients [6], it is plausible that, the chronic inflammatory status in the SCD malaria-positive patients could influence parasite clearance directly, or indirectly, through yet- undetermined action of anti-malarial drugs in SCD patients.

It is possible that the observed PRR differences between the SCD and non-SCD patients reflect intrinsic differences in peripheral blood components and specific variations in cell types between SCD and non-SCD subjects. It is usually assumed that malaria parasites and leucocytes are both distributed evenly on a thick smear; however, it has been shown that in cases of scanty parasitaemia, parasites tend to aggregate in a specific area of a blood smear [54]. This suggests that parasite density estimations based on assumption of a homogenous and, therefore, Poisson-type distribution can result in over-dispersion [55], which may result in over- or under-estimation of parasitaemia.

The observed profiles for the selected haematological indices for the non-SCD subjects (anaemia, thrombocytopaenia and modest neutrophilia-driven leucocytosis), are consistent with those reported for African children with uncomplicated malaria [56, 57]. The corresponding profiles for the SCD subjects are also similar to reported values for SCD subjects [58].

In SCD subjects, elevated platelet counts have been ascribed among others, to absent splenic pooling or functional asplenia [59]. As a reduced platelet count is a feature of malaria infection, the relatively lower mean platelet counts of the SCD-malaria-positive subjects compared with SCD subjects in steady state is likely due to the malarial disease [60, 61]. However, as platelet activation in SCD contributes to a hypercoagulable state, which can result in thrombo-embolic phenomena [62], the implications of these platelet count variations to malaria-related morbidity among SCD subjects are unclear.

## Conclusion

Treatment of children with uncomplicated malaria showed lower PRR and slower parasite clearance in the SCD subjects compared with non-SCD subjects. However, the comparably high day 28 and 42 cure rates in both SCD and non-SCD groups suggest an unlikely effect of this finding on acute malaria management in SCD patients. There were marked variations in leucocyte and platelet counts between acute illness and recovery stages among the SCD patients with malaria. The overall implications of these changes with respect to malaria-related morbidity among SCD patients are uncertain.
